# (2,7-Dimeth­oxy­naphthalen-1-yl)(4-phen­oxy­phen­yl)methanone

**DOI:** 10.1107/S1600536813004820

**Published:** 2013-02-23

**Authors:** Kosuke Sasagawa, Rei Sakamoto, Daichi Hijikata, Noriyuki Yonezawa, Akiko Okamoto

**Affiliations:** a Department of Organic and Polymer Materials Chemistry, Tokyo University of Agriculture & Technology, Koganei, Tokyo 184-8588, Japan

## Abstract

In the title mol­ecule, C_25_H_20_O_4_, the naphthalene and phen­oxy groups are oriented nearly perpendicular with respect to the benzene ring of the benzoyl group, with dihedral angles of 89.61 (5) and 86.13 (6)°, respectively. The crystal structure features C—H⋯O and C—H⋯π inter­actions.

## Related literature

For the formation reactions of aroylated naphthalene compounds *via* electrophilic aromatic substitution of naphthalene derivatives, see: Okamoto & Yonezawa (2009 ▶); Okamoto *et al.* (2011 ▶). For the structures of closely related compounds, see: Hijikata *et al.* (2010 ▶); Nakaema *et al.* (2008 ▶); Sasagawa *et al.* (2013 ▶); Tsumuki *et al.* (2011 ▶, 2012 ▶).
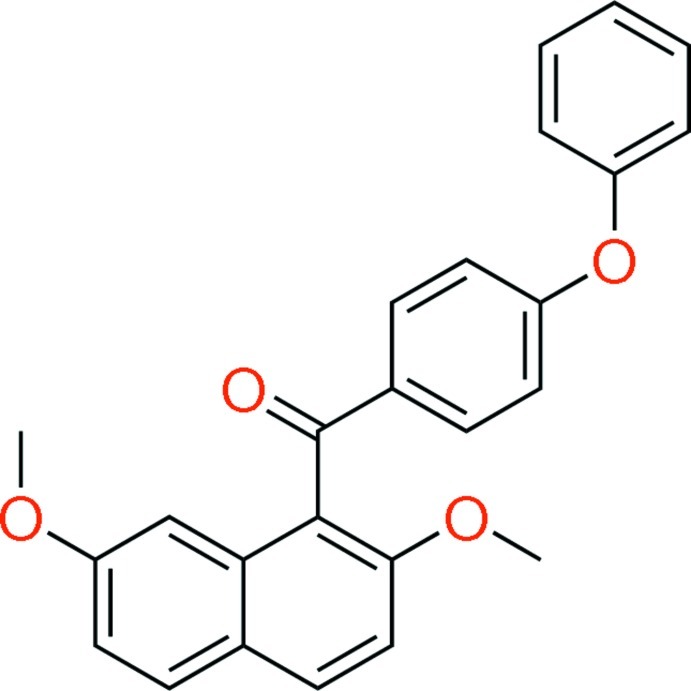


## Experimental

### 

#### Crystal data

C_25_H_20_O_4_
*M* *_r_* = 384.41
Monoclinic, 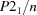

*a* = 10.9512 (2) Å
*b* = 15.8830 (3) Å
*c* = 11.2184 (2) Å
β = 92.460 (1)°
*V* = 1949.51 (6) Å^3^
*Z* = 4
Cu *K*α radiation
μ = 0.71 mm^−1^
*T* = 193 K
0.60 × 0.40 × 0.20 mm

#### Data collection

Rigaku R-AXIS RAPID diffractometer
Absorption correction: numerical (*NUMABS*; Higashi, 1999 ▶) *T*_min_ = 0.674, *T*_max_ = 0.871
35423 measured reflections
3551 independent reflections
3228 reflections with *I* > 2σ(*I*)
*R*_int_ = 0.055

#### Refinement

*R*[*F* ^2^ > 2σ(*F* ^2^)] = 0.037
*wR*(*F* ^2^) = 0.097
*S* = 1.05
3551 reflections
265 parameters
H-atom parameters constrained
Δρ_max_ = 0.21 e Å^−3^
Δρ_min_ = −0.16 e Å^−3^

### 

Data collection: *PROCESS-AUTO* (Rigaku, 1998 ▶); cell refinement: *PROCESS-AUTO*; data reduction: *PROCESS-AUTO*; program(s) used to solve structure: *SHELXS97* (Sheldrick, 2008 ▶); program(s) used to refine structure: *SHELXL97* (Sheldrick, 2008 ▶); molecular graphics: *ORTEPIII* (Burnett & Johnson, 1996 ▶); software used to prepare material for publication: *SHELXL97*.

## Supplementary Material

Crystal structure: contains datablock(s) I, global. DOI: 10.1107/S1600536813004820/gk2553sup1.cif

Structure factors: contains datablock(s) I. DOI: 10.1107/S1600536813004820/gk2553Isup2.hkl

Supplementary material file. DOI: 10.1107/S1600536813004820/gk2553Isup3.cml

Additional supplementary materials:  crystallographic information; 3D view; checkCIF report

## Figures and Tables

**Table 1:** Hydrogen-bond geometry (Å, °) *Cg*1 and *Cg*2 are the centroids of the C20–C25 and C12–C17 benzene rings, respectively.

*D*—H⋯*A*	*D*—H	H⋯*A*	*D*⋯*A*	*D*—H⋯*A*
C21—H21⋯O2^i^	0.95	2.56	3.3738 (17)	143
C19—H19*A*⋯*Cg*1^ii^	0.98	2.74	3.6967 (18)	164
C19—H19*C*⋯*Cg*2^iii^	0.98	2.67	3.6249 (18)	165

Symmetry codes: (i) 
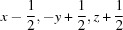
; (ii) 
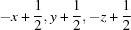
; (iii) 
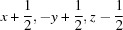
.

**Fig. 1.**
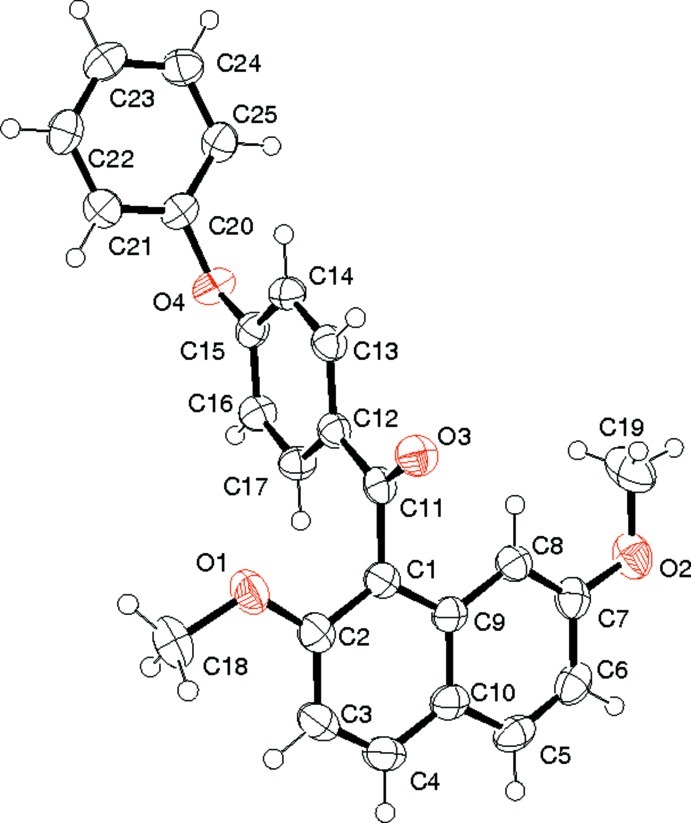
The molecular structure of the title compound and the atom-labeling scheme. Displacement ellipsoids are drawn at the 50% probability level.

**Fig. 2.**
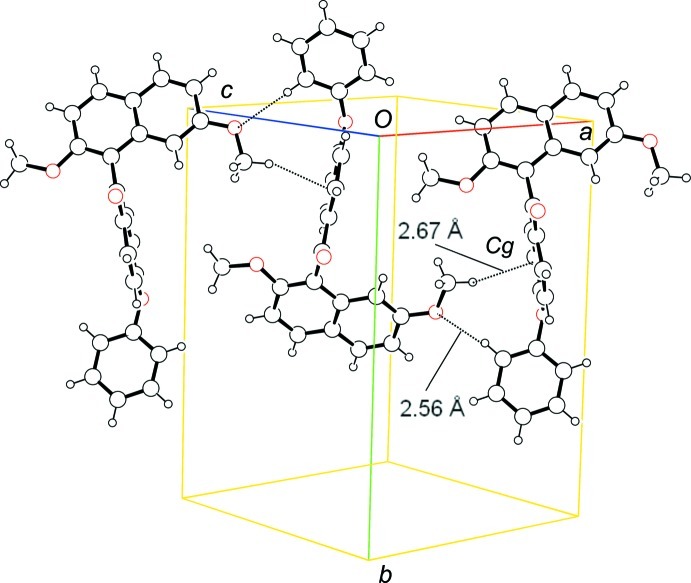
Intermolecular C—H···O interactions between H21 and O2, C—H···π interactions between H19C and Cg [symmetry code: -1/2 + x, 1/2 - y, 1/2 + z; -1/2 + x, 1/2 - y, 1/2 + z] along the a axis (dashed lines).

**Fig. 3.**
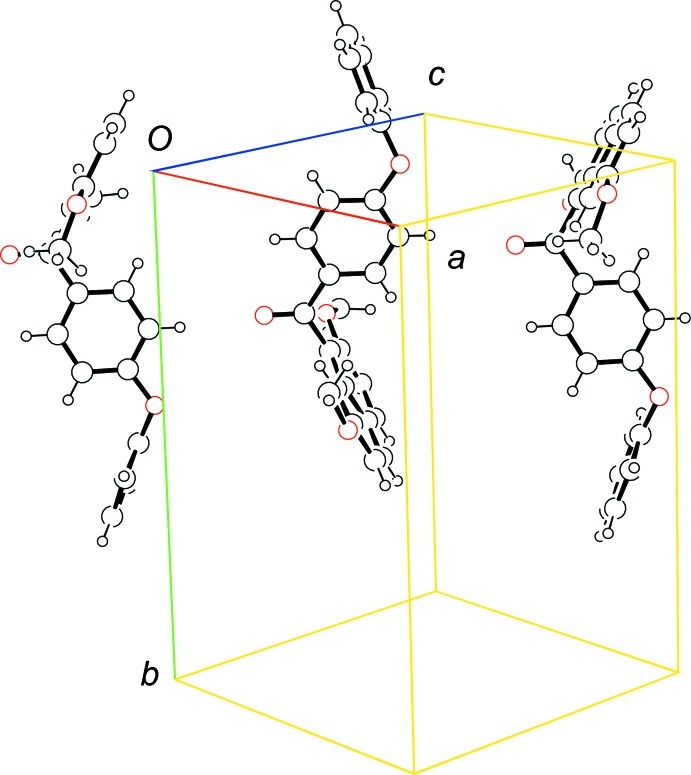
The alignment of the molecules along the c axis.

## supplementary crystallographic information

### Comment

In the course of our study on selective electrophilic aromatic aroylation of the naphthalene ring core, 1-aroylnaphthalene and 1,8-diaroylnaphthalene compounds have proved to be formed regioselectively by the aid of a suitable acidic mediator (Okamoto & Yonezawa, 2009, Okamoto *et al.*, 2011). Recently, we have reported the X-ray crystal structures of 1,8-diaroylated 2,7-dimethoxynaphthalene derivatives such as 1,8-dibenzoyl-2,7-dimethoxynaphthalene (Nakaema *et al.*, 2008) and [2,7-dimethoxy-8-(2-naphthoyl)-naphthalen-1-yl](naphthalen-2-yl)methanone [1,8-bis(2-naphthoyl)-2,7-dimethoxynaphthalene] (Tsumuki *et al.*, 2011).

The aroyl groups in the 1,8-diaroylnaphthalene compounds are almost perpendicular to the naphthalene rings and oriented in opposite directions (*anti*-orientation). On the other hand, we have also clarified another structure of the 1,8-diaroylnaphthalene derivatives, with the two aroyl groups are oriented in the same direction (*syn*-orientation) [2,7-dimethoxy-1,8-bis(4-phenoxybenzoyl)naphthalene; Hijikata *et al.*, 2010].

Moreover, we have reported crystal structures of 1-aroylnapthalene compounds such as (2,7-dimethoxynaphthalen-1-yl)-(4-methoxyphenyl)methanone [1-(4-methoxybenzoyl-2,7-dimethoxynaphthalene) (Sasagawa *et al.*, 2013) and 2,7-dimethoxy-1-(2-naphthoyl)naphthalene (Tsumuki *et al.*, 2012). They have essentially the same non-coplanar structure as the homologous 1,8-diaroylnaphthalenes, *i.e.*, the aroyl group is twisted away from the naphthalene ring.

As a part of our ongoing studies on the molecular structures of these kinds of homologous molecules, the X-ray crystal structure of the title compound, (2,7-dimethoxynaphthalen-1-yl)(4-phenoxyphenyl)methanone, 2,7-dimethoxynaphthalene bearing phenoxybenzoyl group at the 1-position, is discussed in this article.

The molecular structure of the title compound is displayed in Fig 1. The dihedral angle between the best planes of the benzene ring of the internal benzoyl moiety and the naphthalene ring is 89.61 (5) °. In addition, the dihedral angle between the benzene rings of 4-phenoxybenzoyl moiety is 86.13 (6) °.

The ketonic carbonyl moiety (C11═O3) and the internal benzene ring are nearly coplanar [torsion angle O3—C11—C12—C13 = -1.98 (17) °].

In the crystal, two kinds of interactions effectively contribute to stabilization of the molecular packing: (i) C—H···O interaction between the ethereal O atom of the methoxy group at the 7-position of the naphthalene ring and the aromatic H atom at the 2-position of the terminal phenoxy group and (ii) C—H···π interaction between a H atom of the methoxy group at the 7-position of the naphthalene ring and the benzene ring of the internal benzoyl moiety (C21—H21···O2 = 2.56 Å, symmetry code: -1/2+*x*, 1/2-*y*, 1/2+*z*; C19—H19C···*Cg* = 2.67 Å, symmetry code: -1/2+*x*, 1/2-*y*, 1/2+*z*; Fig. 2). Moreover, the molecules are alternately aligned along *c* axis (Fig. 3).

### Experimental

In a 10 ml one-necked flask equipped with a condenser, (2,7-dimethoxynaphthalen-1-yl)-(4-fluorophenyl)methanone (1.0 mmol, 310 mg), phenol (1.0 mmol, 94.1 mg), potassium carbonate (5.0 mmol, 691 mg) and freshly distilled DMAc (2.5 ml) were stirred at 423 K for 6 h. This mixture was poured into 2*M* aqueous HCl (100 ml). The aqueous layer was extracted with ethyl acetate (20 ml × 3). The combined extracts were washed with water followed by washing with brine. The extracts thus obtained were dried over anhydrous MgSO_4_. The solvent was removed under reduced pressure to give a cake (yield 89%). The crude material was purified by column chromatography (silica gel, CHCl_3_) to give the title compound (isolated yield 74%). The isolated product was recrystallized from hexane and CHCl_3_ (3:1 *v*/*v*) to give block-like colorless single-crystals of the title compound.

Spectroscopic Data: ^1^H NMR *δ* (400 MHz, CDCl_3_): 3.74 (3*H*, s), 3.82 (3*H*, s), 6.79 (1*H*, d, *J* = 2.3 Hz), 6.95 (2*H*, d, *J* = 8.7 Hz), 7.01 (1*H*, dd, *J* = 2.3, 7.2 Hz), 7.08 (2*H*, d, *J* = 7.4 Hz), 7.15–7.20 (2*H*, m), 7.39 (2*H*, t, *J* =7.8 Hz), 7.71 (1*H*, d, *J* = 8.7 Hz), 7.83–7.86 (3*H*, m) p.p.m.

^13^C NMR δ (125 MHz, CDCl_3_): 55.22, 56.39, 102.20, 110.25, 117.02, 117.14, 120.30, 121.91, 124.38, 124.68, 129.65, 130.01, 130.82, 131.94, 132.65, 132.99, 154.73, 155.30, 158.79, 162.34, 196.58 p.p.m.

IR (KBr): 1659 (C=O), 1625, 1599, 1511 (Ar), 1239 (OMe) cm^-1^

HRMS (m/*z*): [*M*+H]^+^ calcd. for C_25_H_21_O_4,_ 385.1440, found, 385.1478

m.p. = 409.7–412.2 K

### Refinement

All H atoms were found in a difference map and were subsequently refined as riding atoms, with C—H = 0.95 (aromatic) and 0.98 (methyl) Å with *U*_ĩso_(H) = 1.2 *U*_eq_(C).

### Crystal data

**Table:**

C_25_H_20_O_4_	*F*(000) = 808
*M**_r_* = 384.41	*D*_x_ = 1.310 Mg m^−^^3^
Monoclinic, *P*2_1_/*n*	Cu *K*α radiation, λ = 1.54187 Å
Hall symbol: -P 2yn	Cell parameters from 30559 reflections
*a* = 10.9512 (2) Å	θ = 3.9–68.2°
*b* = 15.8830 (3) Å	µ = 0.71 mm^−^^1^
*c* = 11.2184 (2) Å	*T* = 193 K
β = 92.460 (1)°	Block, colourless
*V* = 1949.51 (6) Å^3^	0.60 × 0.40 × 0.20 mm
*Z* = 4	

### Data collection

**Table:**

Rigaku R-AXIS RAPID diffractometer	3551 independent reflections
Radiation source: fine-focus sealed tube	3228 reflections with *I* > 2σ(*I*)
Graphite monochromator	*R*_int_ = 0.055
Detector resolution: 10.000 pixels mm^-1^	θ_max_ = 68.2°, θ_min_ = 4.8°
ω scans	*h* = −13→13
Absorption correction: numerical (*NUMABS*; Higashi, 1999)	*k* = −18→17
*T*_min_ = 0.674, *T*_max_ = 0.871	*l* = −13→13
35423 measured reflections	

### Refinement

**Table:**

Refinement on *F*^2^	Secondary atom site location: difference Fourier map
Least-squares matrix: full	Hydrogen site location: inferred from neighbouring sites
*R*[*F*^2^ > 2σ(*F*^2^)] = 0.037	H-atom parameters constrained
*wR*(*F*^2^) = 0.097	*w* = 1/[σ^2^(*F*_o_^2^) + (0.0483*P*)^2^ + 0.525*P*] where *P* = (*F*_o_^2^ + 2*F*_c_^2^)/3
*S* = 1.05	(Δ/σ)_max_ < 0.001
3551 reflections	Δρ_max_ = 0.21 e Å^−^^3^
265 parameters	Δρ_min_ = −0.16 e Å^−^^3^
0 restraints	Extinction correction: *SHELXL97* (Sheldrick, 2008), Fc^*^=kFc[1+0.001xFc^2^λ^3^/sin(2θ)]^-1/4^
Primary atom site location: structure-invariant direct methods	Extinction coefficient: 0.0082 (4)

### Special details

**Table:**

Geometry. All e.s.d.'s (except the e.s.d. in the dihedral angle between two l.s. planes) are estimated using the full covariance matrix. The cell e.s.d.'s are taken into account individually in the estimation of e.s.d.'s in distances, angles and torsion angles; correlations between e.s.d.'s in cell parameters are only used when they are defined by crystal symmetry. An approximate (isotropic) treatment of cell e.s.d.'s is used for estimating e.s.d.'s involving l.s. planes.
Refinement. Refinement of *F*^2^ against ALL reflections. The weighted *R*-factor *wR* and goodness of fit *S* are based on *F*^2^, conventional *R*-factors *R* are based on *F*, with *F* set to zero for negative *F*^2^. The threshold expression of *F*^2^ > σ(*F*^2^) is used only for calculating *R*-factors(gt) *etc*. and is not relevant to the choice of reflections for refinement. *R*-factors based on *F*^2^ are statistically about twice as large as those based on *F*, and *R*- factors based on ALL data will be even larger.

### Fractional atomic coordinates and isotropic or equivalent isotropic displacement parameters (Å^2^)

**Table:**

	*x*	*y*	*z*	*U*_iso_*/*U*_eq_	
O1	−0.00193 (9)	0.35588 (7)	0.60358 (8)	0.0457 (3)	
O2	0.50090 (9)	0.46871 (7)	0.23813 (9)	0.0510 (3)	
O3	0.05797 (8)	0.31431 (6)	0.32341 (8)	0.0414 (2)	
O4	0.36378 (8)	0.01835 (6)	0.57454 (9)	0.0410 (2)	
C1	0.15314 (11)	0.39480 (8)	0.47929 (11)	0.0327 (3)	
C2	0.08369 (12)	0.41451 (9)	0.57557 (11)	0.0367 (3)	
C3	0.10230 (13)	0.49068 (9)	0.63775 (12)	0.0420 (3)	
H3	0.0522	0.5049	0.7018	0.050*	
C4	0.19275 (13)	0.54391 (9)	0.60541 (12)	0.0424 (3)	
H4	0.2050	0.5951	0.6481	0.051*	
C5	0.36492 (13)	0.57900 (8)	0.47775 (12)	0.0420 (3)	
H5	0.3788	0.6301	0.5202	0.050*	
C6	0.43770 (13)	0.55914 (9)	0.38729 (12)	0.0423 (3)	
H6	0.5013	0.5963	0.3663	0.051*	
C7	0.41872 (12)	0.48276 (9)	0.32399 (11)	0.0390 (3)	
C8	0.32550 (11)	0.42964 (8)	0.35051 (11)	0.0349 (3)	
H8	0.3124	0.3795	0.3056	0.042*	
C9	0.24795 (11)	0.44941 (8)	0.44540 (11)	0.0328 (3)	
C10	0.26844 (12)	0.52532 (8)	0.51068 (11)	0.0370 (3)	
C11	0.12728 (10)	0.31434 (8)	0.41154 (10)	0.0316 (3)	
C12	0.18985 (10)	0.23641 (8)	0.45420 (10)	0.0310 (3)	
C13	0.16678 (11)	0.16053 (8)	0.39508 (11)	0.0334 (3)	
H13	0.1120	0.1596	0.3271	0.040*	
C14	0.22189 (11)	0.08668 (8)	0.43339 (11)	0.0347 (3)	
H14	0.2046	0.0352	0.3929	0.042*	
C15	0.30315 (11)	0.08859 (8)	0.53203 (11)	0.0325 (3)	
C16	0.32949 (11)	0.16352 (8)	0.59129 (11)	0.0362 (3)	
H16	0.3861	0.1644	0.6578	0.043*	
C17	0.27248 (11)	0.23676 (8)	0.55257 (11)	0.0348 (3)	
H17	0.2897	0.2881	0.5934	0.042*	
C18	−0.06764 (14)	0.36858 (11)	0.70934 (13)	0.0530 (4)	
H18A	−0.0098	0.3730	0.7782	0.064*	
H18B	−0.1227	0.3209	0.7207	0.064*	
H18C	−0.1155	0.4206	0.7017	0.064*	
C19	0.49557 (15)	0.38973 (12)	0.17944 (16)	0.0648 (5)	
H19A	0.4162	0.3837	0.1365	0.078*	
H19B	0.5058	0.3445	0.2385	0.078*	
H19C	0.5610	0.3864	0.1227	0.078*	
C20	0.31156 (11)	−0.06082 (8)	0.55142 (12)	0.0349 (3)	
C21	0.22069 (12)	−0.08914 (9)	0.62315 (12)	0.0403 (3)	
H21	0.1893	−0.0538	0.6829	0.048*	
C22	0.17631 (12)	−0.17021 (9)	0.60612 (13)	0.0439 (3)	
H22	0.1143	−0.1909	0.6550	0.053*	
C23	0.22158 (13)	−0.22106 (9)	0.51863 (13)	0.0450 (3)	
H23	0.1908	−0.2765	0.5075	0.054*	
C24	0.31210 (13)	−0.19118 (9)	0.44695 (13)	0.0446 (3)	
H24	0.3430	−0.2262	0.3865	0.054*	
C25	0.35762 (12)	−0.11038 (9)	0.46320 (12)	0.0401 (3)	
H25	0.4196	−0.0895	0.4143	0.048*	

### Atomic displacement parameters (Å^2^)

**Table:**

	*U*^11^	*U*^22^	*U*^33^	*U*^12^	*U*^13^	*U*^23^
O1	0.0440 (5)	0.0554 (6)	0.0386 (5)	−0.0030 (4)	0.0117 (4)	−0.0039 (4)
O2	0.0424 (5)	0.0667 (7)	0.0443 (6)	−0.0153 (5)	0.0065 (4)	−0.0021 (5)
O3	0.0440 (5)	0.0426 (5)	0.0365 (5)	0.0021 (4)	−0.0089 (4)	−0.0001 (4)
O4	0.0368 (5)	0.0320 (5)	0.0531 (6)	−0.0008 (4)	−0.0097 (4)	0.0021 (4)
C1	0.0341 (6)	0.0335 (7)	0.0302 (6)	0.0057 (5)	−0.0018 (5)	0.0010 (5)
C2	0.0363 (7)	0.0410 (7)	0.0325 (6)	0.0064 (5)	−0.0005 (5)	0.0016 (5)
C3	0.0489 (8)	0.0453 (8)	0.0316 (6)	0.0131 (6)	−0.0001 (6)	−0.0042 (6)
C4	0.0566 (8)	0.0342 (7)	0.0358 (7)	0.0092 (6)	−0.0061 (6)	−0.0048 (5)
C5	0.0509 (8)	0.0297 (7)	0.0441 (7)	0.0006 (6)	−0.0138 (6)	0.0030 (5)
C6	0.0433 (7)	0.0391 (8)	0.0437 (7)	−0.0077 (6)	−0.0100 (6)	0.0103 (6)
C7	0.0361 (7)	0.0468 (8)	0.0336 (6)	−0.0027 (6)	−0.0042 (5)	0.0061 (6)
C8	0.0359 (7)	0.0361 (7)	0.0325 (6)	−0.0006 (5)	−0.0030 (5)	−0.0005 (5)
C9	0.0358 (6)	0.0312 (7)	0.0310 (6)	0.0038 (5)	−0.0049 (5)	0.0026 (5)
C10	0.0445 (7)	0.0307 (7)	0.0349 (6)	0.0066 (5)	−0.0088 (5)	0.0022 (5)
C11	0.0284 (6)	0.0376 (7)	0.0289 (6)	−0.0008 (5)	0.0037 (5)	0.0011 (5)
C12	0.0292 (6)	0.0343 (7)	0.0297 (6)	−0.0016 (5)	0.0030 (5)	−0.0003 (5)
C13	0.0329 (6)	0.0375 (7)	0.0297 (6)	−0.0020 (5)	−0.0013 (5)	−0.0011 (5)
C14	0.0364 (6)	0.0319 (7)	0.0358 (6)	−0.0030 (5)	−0.0002 (5)	−0.0039 (5)
C15	0.0286 (6)	0.0323 (7)	0.0368 (6)	−0.0007 (5)	0.0031 (5)	0.0028 (5)
C16	0.0335 (6)	0.0385 (7)	0.0362 (7)	−0.0009 (5)	−0.0053 (5)	−0.0007 (5)
C17	0.0351 (6)	0.0335 (7)	0.0356 (6)	−0.0022 (5)	−0.0021 (5)	−0.0037 (5)
C18	0.0490 (8)	0.0731 (11)	0.0377 (7)	0.0016 (8)	0.0115 (6)	0.0004 (7)
C19	0.0486 (9)	0.0861 (13)	0.0613 (10)	−0.0172 (9)	0.0207 (8)	−0.0246 (9)
C20	0.0301 (6)	0.0314 (7)	0.0426 (7)	0.0011 (5)	−0.0044 (5)	0.0034 (5)
C21	0.0354 (7)	0.0437 (8)	0.0420 (7)	0.0032 (6)	0.0022 (5)	−0.0008 (6)
C22	0.0355 (7)	0.0472 (8)	0.0492 (8)	−0.0043 (6)	0.0027 (6)	0.0101 (6)
C23	0.0440 (8)	0.0323 (7)	0.0581 (9)	−0.0008 (6)	−0.0056 (6)	0.0057 (6)
C24	0.0469 (8)	0.0363 (8)	0.0508 (8)	0.0072 (6)	0.0035 (6)	−0.0032 (6)
C25	0.0355 (7)	0.0395 (8)	0.0458 (7)	0.0028 (6)	0.0060 (6)	0.0047 (6)

### Geometric parameters (Å, º)

**Table:**

O1—C2	1.3677 (16)	C12—C17	1.3969 (17)
O1—C18	1.4282 (16)	C13—C14	1.3794 (18)
O2—C7	1.3645 (16)	C13—H13	0.9500
O2—C19	1.417 (2)	C14—C15	1.3903 (17)
O3—C11	1.2203 (15)	C14—H14	0.9500
O4—C15	1.3732 (15)	C15—C16	1.3875 (18)
O4—C20	1.4011 (15)	C16—C17	1.3814 (18)
C1—C2	1.3833 (17)	C16—H16	0.9500
C1—C9	1.4174 (18)	C17—H17	0.9500
C1—C11	1.5074 (17)	C18—H18A	0.9800
C2—C3	1.4070 (19)	C18—H18B	0.9800
C3—C4	1.363 (2)	C18—H18C	0.9800
C3—H3	0.9500	C19—H19A	0.9800
C4—C10	1.4068 (19)	C19—H19B	0.9800
C4—H4	0.9500	C19—H19C	0.9800
C5—C6	1.354 (2)	C20—C25	1.3771 (19)
C5—C10	1.419 (2)	C20—C21	1.3816 (18)
C5—H5	0.9500	C21—C22	1.387 (2)
C6—C7	1.416 (2)	C21—H21	0.9500
C6—H6	0.9500	C22—C23	1.380 (2)
C7—C8	1.3670 (18)	C22—H22	0.9500
C8—C9	1.4250 (18)	C23—C24	1.387 (2)
C8—H8	0.9500	C23—H23	0.9500
C9—C10	1.4234 (18)	C24—C25	1.386 (2)
C11—C12	1.4841 (17)	C24—H24	0.9500
C12—C13	1.3935 (17)	C25—H25	0.9500
			
C2—O1—C18	118.00 (11)	C13—C14—C15	119.08 (11)
C7—O2—C19	117.23 (11)	C13—C14—H14	120.5
C15—O4—C20	118.52 (9)	C15—C14—H14	120.5
C2—C1—C9	120.30 (12)	O4—C15—C16	116.29 (11)
C2—C1—C11	119.20 (11)	O4—C15—C14	122.83 (11)
C9—C1—C11	120.50 (11)	C16—C15—C14	120.86 (11)
O1—C2—C1	115.50 (12)	C17—C16—C15	119.33 (11)
O1—C2—C3	123.90 (12)	C17—C16—H16	120.3
C1—C2—C3	120.60 (13)	C15—C16—H16	120.3
C4—C3—C2	119.52 (12)	C16—C17—C12	120.91 (12)
C4—C3—H3	120.2	C16—C17—H17	119.5
C2—C3—H3	120.2	C12—C17—H17	119.5
C3—C4—C10	121.96 (13)	O1—C18—H18A	109.5
C3—C4—H4	119.0	O1—C18—H18B	109.5
C10—C4—H4	119.0	H18A—C18—H18B	109.5
C6—C5—C10	121.60 (13)	O1—C18—H18C	109.5
C6—C5—H5	119.2	H18A—C18—H18C	109.5
C10—C5—H5	119.2	H18B—C18—H18C	109.5
C5—C6—C7	119.78 (13)	O2—C19—H19A	109.5
C5—C6—H6	120.1	O2—C19—H19B	109.5
C7—C6—H6	120.1	H19A—C19—H19B	109.5
O2—C7—C8	125.03 (13)	O2—C19—H19C	109.5
O2—C7—C6	114.01 (12)	H19A—C19—H19C	109.5
C8—C7—C6	120.96 (13)	H19B—C19—H19C	109.5
C7—C8—C9	120.07 (12)	C25—C20—C21	121.85 (13)
C7—C8—H8	120.0	C25—C20—O4	119.14 (12)
C9—C8—H8	120.0	C21—C20—O4	118.87 (12)
C1—C9—C10	118.78 (12)	C20—C21—C22	118.62 (13)
C1—C9—C8	122.17 (11)	C20—C21—H21	120.7
C10—C9—C8	119.02 (12)	C22—C21—H21	120.7
C4—C10—C5	122.68 (13)	C23—C22—C21	120.48 (13)
C4—C10—C9	118.78 (13)	C23—C22—H22	119.8
C5—C10—C9	118.53 (12)	C21—C22—H22	119.8
O3—C11—C12	121.56 (11)	C22—C23—C24	119.99 (13)
O3—C11—C1	120.38 (11)	C22—C23—H23	120.0
C12—C11—C1	118.05 (10)	C24—C23—H23	120.0
C13—C12—C17	118.53 (11)	C25—C24—C23	120.17 (13)
C13—C12—C11	119.74 (11)	C25—C24—H24	119.9
C17—C12—C11	121.73 (11)	C23—C24—H24	119.9
C14—C13—C12	121.28 (11)	C20—C25—C24	118.88 (13)
C14—C13—H13	119.4	C20—C25—H25	120.6
C12—C13—H13	119.4	C24—C25—H25	120.6
			
C18—O1—C2—C1	173.66 (12)	C2—C1—C11—O3	93.21 (15)
C18—O1—C2—C3	−6.68 (19)	C9—C1—C11—O3	−87.20 (15)
C9—C1—C2—O1	−177.41 (10)	C2—C1—C11—C12	−87.71 (14)
C11—C1—C2—O1	2.18 (17)	C9—C1—C11—C12	91.87 (13)
C9—C1—C2—C3	2.93 (18)	O3—C11—C12—C13	−1.97 (18)
C11—C1—C2—C3	−177.49 (11)	C1—C11—C12—C13	178.96 (11)
O1—C2—C3—C4	177.88 (12)	O3—C11—C12—C17	177.75 (11)
C1—C2—C3—C4	−2.48 (19)	C1—C11—C12—C17	−1.31 (17)
C2—C3—C4—C10	0.3 (2)	C17—C12—C13—C14	1.26 (18)
C10—C5—C6—C7	−0.58 (19)	C11—C12—C13—C14	−179.00 (11)
C19—O2—C7—C8	−5.8 (2)	C12—C13—C14—C15	−0.83 (18)
C19—O2—C7—C6	173.69 (13)	C20—O4—C15—C16	156.41 (11)
C5—C6—C7—O2	−177.64 (12)	C20—O4—C15—C14	−25.21 (17)
C5—C6—C7—C8	1.92 (19)	C13—C14—C15—O4	−178.65 (11)
O2—C7—C8—C9	177.58 (11)	C13—C14—C15—C16	−0.34 (18)
C6—C7—C8—C9	−1.93 (19)	O4—C15—C16—C17	179.47 (11)
C2—C1—C9—C10	−1.23 (17)	C14—C15—C16—C17	1.05 (19)
C11—C1—C9—C10	179.19 (10)	C15—C16—C17—C12	−0.60 (19)
C2—C1—C9—C8	176.92 (11)	C13—C12—C17—C16	−0.53 (18)
C11—C1—C9—C8	−2.66 (17)	C11—C12—C17—C16	179.74 (11)
C7—C8—C9—C1	−177.51 (11)	C15—O4—C20—C25	102.71 (14)
C7—C8—C9—C10	0.64 (18)	C15—O4—C20—C21	−81.52 (15)
C3—C4—C10—C5	−178.39 (12)	C25—C20—C21—C22	0.82 (19)
C3—C4—C10—C9	1.32 (19)	O4—C20—C21—C22	−174.82 (11)
C6—C5—C10—C4	179.03 (12)	C20—C21—C22—C23	−0.5 (2)
C6—C5—C10—C9	−0.67 (18)	C21—C22—C23—C24	0.0 (2)
C1—C9—C10—C4	−0.86 (17)	C22—C23—C24—C25	0.2 (2)
C8—C9—C10—C4	−179.07 (11)	C21—C20—C25—C24	−0.6 (2)
C1—C9—C10—C5	178.86 (11)	O4—C20—C25—C24	175.01 (11)
C8—C9—C10—C5	0.65 (17)	C23—C24—C25—C20	0.1 (2)

### Hydrogen-bond geometry (Å, º)

Cg1 and Cg2 are the centroids of the C20-C25 and C12-C17 benzene rings, respectively.

**Table:**

*D*—H···*A*	*D*—H	H···*A*	*D*···*A*	*D*—H···*A*
C21—H21···O2^i^	0.95	2.56	3.3738 (17)	143
C19—H19*A*···*Cg*1^ii^	0.98	2.74	3.6967 (18)	164
C19—H19*C*···*Cg*2^iii^	0.98	2.67	3.6249 (18)	165

Symmetry codes: (i) *x*−1/2, −*y*+1/2, *z*+1/2; (ii) −*x*+1/2, *y*+1/2, −*z*+1/2; (iii) *x*+1/2, −*y*+1/2, *z*−1/2.

## References

[bb1] Burnett, M. N. & Johnson, C. K. (1996). *ORTEPIII* Report ORNL-6895. Oak Ridge National Laboratory, Tennessee, USA.

[bb2] Higashi, T. (1999). *NUMABS* Rigaku Corporation, Tokyo, Japan.

[bb3] Hijikata, D., Takada, T., Nagasawa, A., Okamoto, A. & Yonezawa, N. (2010). *Acta Cryst.* E**66**, o2902–o2903. 10.1107/S1600536810042170 PMC3009209 21589079

[bb4] Nakaema, K., Watanabe, S., Okamoto, A., Noguchi, K. & Yonezawa, N. (2008). *Acta Cryst.* E**64**, o807. 10.1107/S1600536808007009 PMC2961268 21202298

[bb5] Okamoto, A., Mitsui, R., Oike, H. & Yonezawa, N. (2011). *Chem* *Lett* **40**, 1283–1284.

[bb6] Okamoto, A. & Yonezawa, N. (2009). *Chem. Lett.* **38**, 914–915.

[bb7] Rigaku (1998). *PROCESS-AUTO* Rigaku Corporation, Tokyo, Japan.

[bb8] Sasagawa, K., Sakamoto, R., Hijikata, D., Okamoto, A. & Yonezawa, N. (2013). *Acta Cryst.* E**69**, o363. 10.1107/S1600536813003218 PMC3588548 23476553

[bb9] Sheldrick, G. M. (2008). *Acta Cryst.* A**64**, 112–122. 10.1107/S0108767307043930 18156677

[bb10] Tsumuki, T., Hijikata, D., Okamoto, A., Oike, H. & Yonezawa, N. (2011). *Acta Cryst.* E**67**, o2095. 10.1107/S1600536811028054 PMC3213537 22091114

[bb11] Tsumuki, T., Isogai, A., Nagasawa, A., Okamoto, A. & Yonezawa, N. (2012). *Acta Cryst.* E**68**, o2595. 10.1107/S1600536812033545 PMC3415031 22905018

